# Repeated inoculation with fresh rumen fluid before or during weaning modulates the microbiota composition and co-occurrence of the rumen and colon of lambs

**DOI:** 10.1186/s12866-020-1716-z

**Published:** 2020-02-07

**Authors:** Shaobo Yu, Guangyu Zhang, Zhibo Liu, Peng Wu, Zhongtang Yu, Jiakun Wang

**Affiliations:** 1 Institute of Dairy Science, College of Animal Sciences, Zhejiang University, Hangzhou, China; 2 Department of Animal Sciences, The Ohio State University, Columbus, OH USA

**Keywords:** Bacterial colonization, Bacterial co-occurrence, Lambs, Rumen fluid inoculation

## Abstract

**Background:**

Many recent studies have gravitated towards manipulating the gastrointestinal (GI) microbiome of livestock to improve host nutrition and health using dietary interventions. Few studies, however, have evaluated if inoculation with rumen fluid could effectively reprogram the development of GI microbiota. We hypothesized that inoculation with rumen fluid at an early age could modulate the development of GI microbiota because of its low colonization resistance.

**Results:**

In this study, we tested the above hypothesis using young lambs as a model. Young lambs were orally inoculated repeatedly (four times before or twice during gradual weaning) with the rumen fluid collected from adult sheep. The oral inoculation did not significantly affect starter intake, growth performance, or ruminal fermentation. Based on sequencing analysis of 16S rRNA gene amplicons, however, the inoculation (both before and during weaning) affected the assemblage of the rumen microbiota, increasing or enabling some bacterial taxa to colonize the rumen. These included operational taxonomic units (OTUs) belonging to *Moryella*, *Acetitomaculum*, *Tyzzerella* 4, *Succiniclasticum*, *Prevotella* 1, *Lachnospiraceae*, *Christensenellaceae R-7* group, *Family XIII* AD3011, and *Bacteroidales S24–7* corresponding to inoculation before weaning; and OTUs belonging to *Succiniclasticum*, *Prevotellaceae* UCG-003, *Erysipelotrichaceae* UCG-004, *Prevotella* 1, *Bacteroidales S24–7* gut group uncultured bacterium, and candidate *Family XIII* AD3011 corresponding to inoculation during weaning. Compared to the inoculation during weaning, the inoculation before weaning resulted in more co-occurrences of OTUs that were exclusively predominant in the inoculum. However, inoculation during weaning appeared to have more impacts on the colonic microbiota than the inoculation before weaning. Considerable successions in the microbial colonization of the GI tracts accompanied the transition from liquid feed to solid feed during weaning.

**Conclusions:**

Repeated rumen fluid inoculation during early life can modulate the establishment of the microbiota in both the rumen and the colon and co-occurrence of some bacteria. Oral inoculation with rumen microbiota may be a useful approach to redirect the development of the microbiota in both the rumen and colon.

## Background

A mature rumen harbors a microbiota of high diversity, density, and complexity [1, 2]. The rumen microbiota enables digestion and utilization of the forage and other plant materials that non-ruminants cannot digest or utilize and production of volatile fatty acid (VFAs) and microbial crude protein (MCP), which serve as the primary energy and protein sources, respectively, for cattle, sheep, and other ruminants [3, 4]. Additionally, gastrointestinal microbiota is also implicated in host nutrient harvest and health, primarily in the GI tracts. Therefore, many research efforts have been made to improve feed utilization efficiency and animal health by modulating the rumen and GI microbiotas (broadly speaking, GI microbiota can also refer to the rumen microbiota, but to avoid confusion, GI microbiota only refer to the microbiota in the small and large intestines in this paper) through dietary interventions [5, 6]. Unfortunately, few dietary interventions, including monensin [7] and phytochemicals [8], have achieved consistent or persistent efficacy. This is because the GI microbiota of adults is rather stable and resilient [9], and the dietary interventions have to be within a “safe” range that will not harm the host animals or the GI microbiota. Furthermore, dietary interventions need to be continuous, which would increase production costs. Thus, other approaches including microbiota intervention are sought after in recent years.

It is a straightforward approach to modulate the GI microbiota of animals by orally inoculating their GI tract with the GI microbiota from donor animals exhibiting desired health or production phenotypes. A number of studies have attempted to augment rumen function(s) by inoculating rumen microbes but reported mixed results. Indeed, when strains of *Ruminococcus albus* and *Ruminococcus flavefaciens*, all of which were isolated from the rumen, were inoculated into the rumen of adult sheep, the inoculants failed to persist or improve the intended fiber digestion [10]. *Megasphaera elsdenii* inoculation to the rumen of Holstein heifers suffering from subacute ruminal acidosis (SARA) did not increase the abundance of *M. elsdenii* or alleviate the SARA conditions although it altered the rumen VFA profiles [11–13]. Inoculation with whole rumen contents from non-milkfat-depressed cows only slightly accelerated the recovery of de novo synthesis of fatty acids in milk fat-depressed cows [14]. Even the exchange of nearly all the rumen contents between two dairy cows only achieved transient alteration of their rumen microbiota and lactation performance [15]. When 70% of the rumen contents of steers were replaced by that of basins, which are more efficient in forage digestion, the recipient steers did not show the anticipated improvement in fiber digestion although the steers exhibited improved nitrogen utilization [16]. Host specificity, colonization resistance, and microbiota resilience inherent of the climax GI microbiota [9, 15, 17] may explain the difficulties in modulating the GI microbiota of adult ruminants.

Early life, especially before weaning, is a critical period during which the developmental plasticity can be profoundly affected with long-term consequences [18]. This also applies to the development of the GI microbiota and its function [19] and has been demonstrated by Distel et al. [20, 21] who showed that dietary treatments had more profound and lasting effects in young lambs than in adult sheep. It was also shown that early dietary experience increased the preference for and the consumption of those feeds by animals [22]. Calves and lambs are thought to be born with a sterile rumen [23, 24], but the rumen is rapidly colonized by different microbes during and after birth [25]. Driven by animal growth and diet, the rumen microbiota increases in density, diversity, and complexity, with some bacteria appearing in the rumen only after the animals start to consume starter or forage while some microbes being more abundant generally in the adult rumen [26–29]. From an ecological perspective, the simple and less diverse GI microbiota of newborn animals is more receptive to exogenous inoculation because it has less colonization resistance. Thus, we hypothesized that ruminal microbiota transplantation (RMT) with mature rumen microbiota to newborn lambs could alter the GI microbiota of the latter and improve their feed digestibility and growth performance during the weaning period, eventually leading to a better foundation for feed adaption and post-weaning growth. In this study, we tested the above hypothesis by investigating how RMT from mature sheep to young lambs would modulates the microbiota of the rumen and colon of the latter. Because weaning has considerable effects on the rumen and GI microbiotas [30] and the growth rate of calves or lambs [31, 32] due to the transition from liquid to solid feed and the weaning stress, RMT was done both before and during weaning for comparisons.

## Results

### Animal growth, GI tract growth and fermentation, and fecal scores

**Fig. 1:**
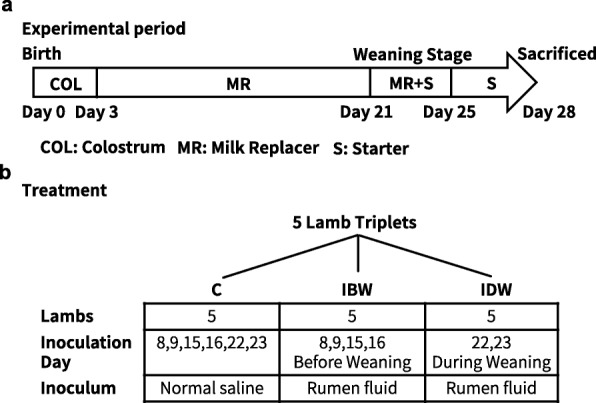
Experimental design. Study design showing the time frame of the experiment (**a**) and the treatment groups (**b**). Each group had five lambs from five different triplets. Solid feed was supplied from day 21, and milk replacer was completely withdrawn at day 25. C: Control receiving mock inoculation with saline; IBW: Inoculation before weaning; IDW: Inoculation during weaning

The animal experiment design was illustrated in Fig. 1. Both the lambs inoculated before weaning (IBW) and the lambs inoculated during weaning (IDW) had a numerically greater accumulated starter intake than lambs mock-inoculated with 20 ml normal saline (6 inoculations, the control (C) group, Table 1). Compared to the control lambs, the IBW lambs had numerically greater average daily gains (ADG) before weaning and smaller negative ADG (− 24.3 vs. -27.1 g/d) since the weaning started, while the IDW lambs had the smallest negative ADG (− 12.1 g/d). Both ADG and accumulated starter intake were similar among the triplet blocks. The molar proportions of individual VFAs in the rumen contents were not influenced by the inoculation except that of valerate being significantly higher in the IDW group (*P* = 0.03) and that of isovalerate being significantly higher in the IBW and the IDW lambs than the control (*P* = 0.01). Acetate to propionate (A: P) ratio, pH, and concentration of ammonia nitrogen (NH_3_-N) in the rumen did not differ among the lamb groups or the triplet blocks (Table 2). In the colon content samples, branch chain VFAs were not detectable, while the concentration of total VFAs or the molar proportion of individual VFAs were not affected by the inoculation or triplet blocks. The inoculation did not affect the empty weights (normalized against bodyweight) of each of the four rumen compartments or the small and large intestines (Table 1). However, the IBW and the IDW lambs had a significantly lower (*P* = 0.01) empty weight of the colon compared to the control group (Table 1). The length of the small and the large intestines did not differ among the three lamb groups or the triplet blocks. Scouring days and fecal scores, either before or after the feed transition during weaning, were not different (*P* > 0.05) among the three inoculation groups (Additional file 1: Table S1).

**Table 1:** Growth performance and measurement of GI tracts

	Inoculation (*n* = 5)^a^			SEM	*P*-value	
	Control	IBW	IDW		Inoculation	Triplets^c^
Performance						
Body weight (kg)	3.44	3.34	3.47	0.06	0.74	0.82
Accumulated starter intake (g)	101.43	104.63	124.59	16.15	0.75	0.08
ADG (g/d):						
Overall (D7–28)	35.02	39.97	37.89	1.79	0.55	0.39
D 7–14	55.00	61.43	50.71	3.25	0.32	0.14
D 14–21	77.14	82.86	75.00	5.02	0.96	0.38
D 21–28	−27.14	−24.29	−12.14	4.42	0.21	0.08
Weight of GI segment (g/kg BW^b^)						
Rumen	13.45	10.29	12.23	0.97	0.51	0.66
Reticulum	2.95	2.53	2.88	0.24	0.53	0.02
Omasum	1.44	1.44	1.31	0.11	0.90	0.74
Abomasum	6.88	7.37	7.79	0.22	0.31	0.56
Small intestine	28.88	27.29	29.87	0.90	0.43	0.20
Colon+Cecum	12.89	9.32	10.13	0.71	0.05	0.15
Colon	8.30^d^	4.66^b^	5.16^b^	0.58	0.01	0.11
Length of intestinal segments (cm/kg BW^b^)						
Small intestine	183.86	181.13	181.10	4.09	0.95	0.91
Colon+Cecum	36.81	41.70	35.76	3.09	0.22	0.51

^a^: *IBW* Inoculation before weaning, *IDW* Inoculation during weaning

^b^: Body weight before euthanization

^c^: Lambs from same triplets were considered as blocks factor

^d^: Values with different letters within a row were significantly (or significant trend) different according to pairwise comparison

**Table 2:** Fermentation parameters of the rumen and colon content of the lambs

	Inoculation (*n* = 5)^a^			SEM	*P*-value	
	Control	IBW	IDW		Inoculation	Triplets^b^
Rumen fermentation parameters						
pH	5.79	5.78	5.69	0.10	0.90	0.21
Total VFA (mmol/g content)	56.32	55.67	63.64	3.81	0.60	0.23
Acetate (% Total VFA)	63.31	65.49	64.18	1.80	0.83	0.12
Propionate (% Total VFA)	23.72	19.33	19.54	1.10	0.30	0.69
Isobutyrate (% Total VFA)	0.95	1.65	0.62	0.20	0.17	0.98
Butyrate (% Total VFA)	8.13	8.97	11.32	1.09	0.50	0.42
Isovalerate (% Total VFA)	0.90^c^	2.32^a^	1.62^b^	0.23	0.01	0.02
Valerate (% Total VFA)	2.14^b^	2.25^b^	3.60^a^	0.27	0.03	0.28
Acetate:Propionate	2.79	3.65	3.46	0.34	0.58	0.35
NH_3_-N (mg/g content)	0.22	0.30	0.43	0.07	0.24	0.05
Colon fermentation parameters						
Total VFA (mmol/g content)	57.73	47.34	44.42	5.96	0.64	0.33
Acetate (% TotalVFA)	73.30	78.72	72.13	1.24	0.09	0.63
Propionate (% Total VFA)	19.22	15.73	21.36	1.09	0.16	0.74
Butyrate (% Total VFA)	7.48	5.55	6.51	0.39	0.20	0.83
Acetate:Propionate	3.99	5.27	3.47	0.33	0.10	0.65
NH_3_-N (mg/g content)	2.73	1.48	1.19	0.44	0.34	0.39

^a^: *IBW* Inoculation before weaning, *IDW* Inoculation during weaning

^b^: Lambs from same triplets were considered as blocks factor

### Bacterial composition and diversity of the inocula and the rumen contents of the lambs

In total, 1,414,108 quality-checked sequences were clustered into 612 OTUs at 97% sequence similarity with an average Good’s coverage of 99.82 ± 0.01% in all the samples. The numbers of observed OTUs, Chao1 richness estimate, and Shannon diversity index of the microbiota were all significantly higher (*P* < 0.01) in the inocula than in the rumen contents of the lambs (Table 3). *Firmicutes* (39.34 ± 1.96%), *Bacteroides* (45.90 ± 2.34%), and *Proteobacteria* (4.85 ± 0.68%) were the most predominant phyla in all the rumen and the colon samples (Additional file 2: Figure S1). *Bacteroides*, *Prevotella* 1, *Acetitiomaculum*, *Butyrivibrio*-2, and *Lachnospiraceae* NK3A20 were the common genera dominated in both the inoculum microbiota and the rumen microbiota of the lambs (Additional file 2: Figure S1a). Comparisons of the inoculum microbiota and the rumen microbiota of the control lambs revealed 47 genera that were significantly more predominant in the inoculum (referred to as inoculum-predominant genera) than in the rumen of the control lambs. They included the uncultured *BS11* gut group (within *Bacteroidales*), the uncultured *RF16* rumen group (*Bacteroidales*), *Prevotellaceae* UCG-001, *Prevotellaceae* UCG-003, the *Rikenellaceae RC9* gut group, the *Christensenellaceae R-7* group, *Pseudobutyrivibrio*, the *Ruminococcaceae* NK4A214 group, *Ruminococcaceae* UCG-011, and others. *Mogibacterium*, *Sharpea,* and *Treponema* 2 appeared to be the genera significantly more predominant in the rumen of control lambs than in the inocula. At a higher phylogenetic resolution, 271 OTUs exhibited a significant difference in relative abundance between the inocula and the rumen of the control lambs. The OTUs that were more predominant in the inocula (referred to as inoculum-predominant OTUs) were assigned to the *Bacteroidales BS11* gut group, the *Bacteroidales S24–7* gut group, the genera *Prevotella* 1 and *Prevotellaceae* UCG-001, the *Rikenellaceae RC9* gut group, and the *Christensenellaceae R-7* group. Only 6 OTUs were more predominant in the rumen of the control lambs than in the inocula, and these OTUs were assigned to the genera *Sharpea*, *Treponema* 2, and *Prevotella* 1.

**Table 3:** α-Diversity measurements of the microbiota in the rumen and colon content of different inoculation groups

	Inoculum (*n* = 6)	Inoculation (*n* = 5)^a^			SEM	*P*-value		
		Control	IBW	IDW		Inoculum vs. lambs^b^	Inoculation	Triplets^c^
Rumen content								
OTUs	571^d^	507^b^	477^b^	477^b^	14.5	0.008	0.347	0.039
Chao1	588^a^	545^b^	512^b^	521^b^	12.6	0.016	0.305	0.016
Shannon	7.63^a^	5.19^b^	5.87^b^	5.25^b^	0.3	0.004	0.340	0.678
Colon content								
OTUs		411	397	401	19.1		0.902	0.003
Chao1		482	452	435	19.6		0.514	0.039
Shannon		5.38	5.12	5.62	0.21		0.659	0.224

^a^: *IBW* Inoculation before weaning, *IDW* Inoculation during weaning

^b^: Inoculum was compared to all the lamb groups by the Kruskal-Wallis test

^c^: Lambs from same triplets were considered as blocks factor

^d^: Values with different letters within a row were significantly different according to pairwise comparison

### Effect of the inoculation on the rumen and the colon microbiota of the lambs

**Fig. 2:**
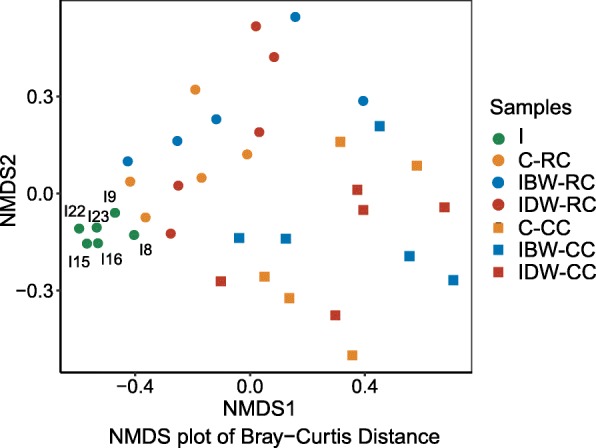
NMDS plot based on Bray-Curtis dissimilarities of all samples. NMDS plot with a stress value of 0.1225. Rumen microbiota (circle) and colon microbiota (square) are separated with a *P* < 0.01; the inoculum microbiota and the rumen microbiota of the lamb groups are also separated with a *P* < 0.01; but the rumen microbiota of the three inoculation groups are not separated with statistical support (*P* > 0.05). I8, I9, I15, I16, I22, I23: Inoculum in different inoculation time; RC: Rumen content; CC: Colon content; IBW: Inoculation before weaning; IDW: Inoculation during weaning

**Fig. 3:**
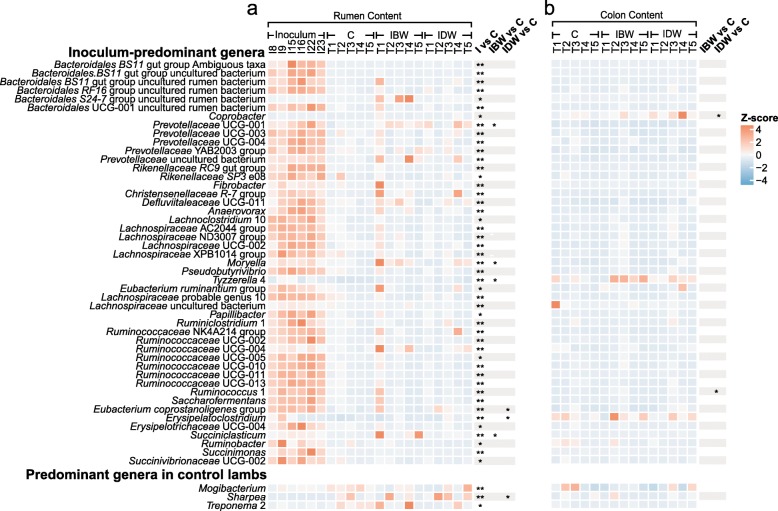
Occurrence of inoculum-predominant bacterial genera in the inoculum, and in the rumen (**a**) and the colon (**b**) of three lamb groups after inoculation. The 50 genera with significant difference in relative abundance among the lamb groups were plotted. The heatmap scale indicates the normalized relative abundance (Z-scores) of each genus. C: Control; IBW: Inoculation before weaning; IDW: Inoculation during weaning. *: *P* < 0.05; **: *P* < 0.01

The overall microbiota of the six inocula did not significantly differ (Fig. 2, Additional file 1: Table S2). The inoculation, either before or during weaning, did not affect any of the α-diversity measurements of the rumen microbiota in the lambs. Both the Chao1 richness estimate and the numbers of observed OTUs of the rumen microbiota of the lambs were significantly influenced by triplets blocks (*P* < 0.05, Table 3). Bray-Curtis dissimilarity of either the ruminal or colonic microbiota was not significantly different among the control, IBW, and IDW (Additional file 1: Table S3), but the inoculum microbiota, the rumen microbiota, and the colon microbiota of the lambs were clustered separately (Fig. 2) with statistical significance (*P* < 0.01). After the inoculation, the genera *Prevotellaceae* UCG-001, *Moryella*, *Succiniclasticum*, and *Tyzzerella* 4 were significantly increased in the rumen of the IBW lambs, and the inoculation did not alter the microbiota in the colon of the IBW group. *Erysipelatoclostridium*, *Eubacterium coprostanoligenes*, and *Sharpea* were increased in the rumen of the IDW group compared to the control group after inoculation (Fig. 3a, Additional file 1: Table S4). The genera *Coprobacter* and *Ruminococcus*-1 increased in the colon contents of the IDW lambs (Fig. 3b, Additional file 1: Table S5).

**Fig. 4:**
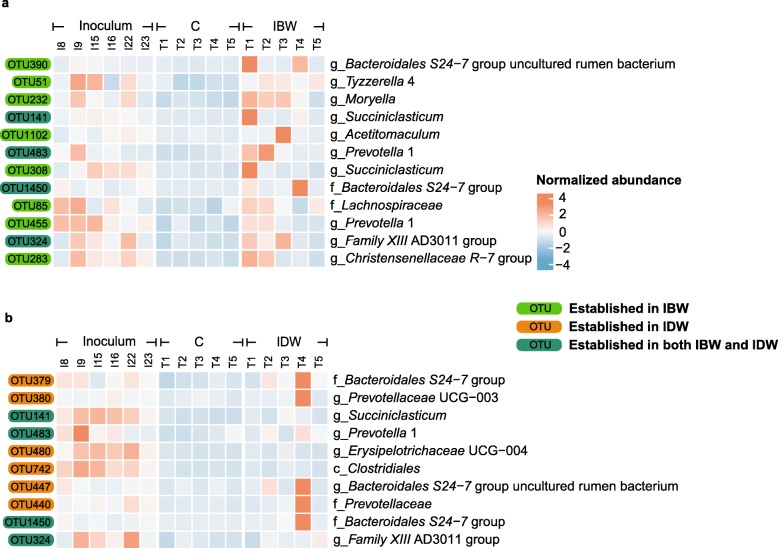
Occurrence of bacterial OTUs in the inoculum and the rumen of the control and the IBW lambs (**a**) or in the inoculum and the rumen of the control and the IDW lambs (**b**). **a** The relative abundance of the OTUs was significantly higher in IBW than in the control; **b** The relative abundance of the OTUs was significantly higher in IDW than in the control. C: Control; IBW: Inoculation before weaning; IDW: Inoculation during weaning

Of the inoculum-predominant OTUs, 12 were established in the rumen of IBW lambs, and they were assigned to the genera *Moryella*, *Acetitomaculum*, *Tyzzerella* 4, *Succiniclasticum*, *Prevotella* 1, *Lachnospiraceae*, the *Christensenellaceae R-7* group, the *Family XIII* AD3011 group, and the *Bacteroidales S24–7* group (Fig. 4a). Ten of the inoculum-predominant OTUs were established in the rumen of IDW lambs, and they were assigned to the genera *Sharpea*, *Succiniclasticum*, *Prevotellaceae* UCG-003, *Erysipelotrichaceae* UCG-004, *Prevotella* 1, the uncultured *S24–7* rumen bacterium (*Bacteroidales*), and the *Family XIII* AD3011 group (Fig. 4b). Among these OTUs, one OTU each belonging to the genus *Succiniclasticum* (OTU141), the *Family XIII* AD3011 group (OTU324), the genus *Prevotella 1* (OTU483), and *Bacteroidales S24–7* group (OTU1450) was established in the rumen of both the IBW and the IDW groups. Several OTUs were detected in the inocula and the rumen of the IBW or the IDW lambs but not the rumen of the control lambs (detected samples > 2 in 5, Additional file 3: Figure S2). For the sake of clarity, these OTUs were referred to as inoculation-promoted taxa. Three OTUs belonging to the *Rikenellaceae RC9* gut group were detected in the rumen of the IBW lambs, while two OTUs belonging to the *Bacteroidales BS11* gut group were present in the rumen of the IDW lambs. More OTUs were detected in the rumen of both the IBW and the IDW lambs than those found only in either the IBW or the IDW group (Additional file 3: Figure S2).

### Co-occurrence of the OTUs established in the rumen of the lambs and the OTUs of the inocula

**Fig. 5:**
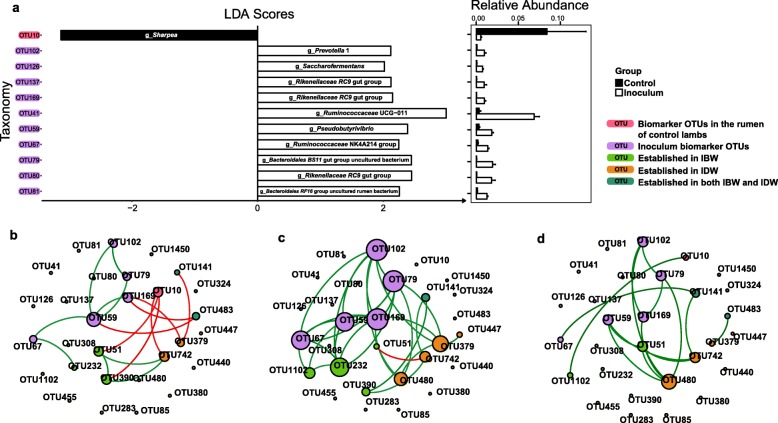
Biomarker OTUs of the inocula and their co-occurrence with the established OTUs after inoculation in the rumen of the lambs. **a** LDA scores and relative abundance of the biomarker OTUs; **b**, **c**, **d** Co-occurrence networks in the rumen the control lambs, IBW lambs, and IDW lambs, respectively. Significant coefficients (> 0.8) are illustrated, with red lines indicating negative correlation, while green lines indicating positive correlation

The LEfSe analysis of the inoculum microbiota and the rumen microbiota of the control lambs identified OTUs that were indicative of each rumen microbiota. These “biomarker” OTUs included ten OTUs belonging to the genera *Prevotella* 1, the *Rikenellaceae RC9* gut group, *Pseudobutyrivibrio*, the *Ruminococcaceae* NK4A214 group, the uncultured *BS11* gut group (*Bacteroidales*), the uncultured *RF16* rumen group (*Bacteroidales*), *Saccharofermentans*, and *Ruminococcaceae* UCG-011, all of which were enriched in the inocula, and only one OTU belonging to the genus *Sharpea*, which was more predominant in the rumen microbiota of the control lambs (Fig. 5a). The network analysis of co-occurrence between the OTUs established in the rumen of the control lambs (i.e., OTU379 and OTU483) and the biomarker OTUs (i.e., OTU59 and OTU169) of the inoculum microbiota revealed several negative co-occurrence relationships (Fig. 5b). However, the IBW group (Fig. 5c) had a greater average node degree (2.07) in the co-occurrence network than the control (1.10) or the IDW (1.03) groups (Fig. 5b, d). Most of the biomarker OTUs of inoculum microbiota and the established OTUs in the inoculated lambs (both the IBW or the IDW), such as OTU232 and OTU1102, showed a positive co-occurrence relationship (Fig. 5c). The two OTUs established in the rumen of the IDW lambs (i.e., OTU480 and OTU742) and the biomarker OTUs of the inoculum microbiota also exhibited a positive co-occurrence relationship (Fig. 5d).

### Compositional changes of the colonic microbiota of the lambs after inoculation

Overall, the rumen fluid inoculation had less impact on the colonic microbiota than on the rumen microbiota, but some of the bacterial genera were affected by the inoculation, both before and during weaning (Fig. 3). Inoculation before weaning increased the relative abundance of *Veillonella* and *Escherichia/Shigella*, while inoculation during weaning expanded the relative abundance of *Veillonella*, *Ruminococcus* 1, *Anaerofilum*, and *Coprobacter* (Additional file 1: Tables S3 and S4). None of the inoculum-predominant genera increased its relative abundance in the colon of the lambs when the inoculation was done before weaning. OTU157 belonging to *Butyrivibrio* 2 was only detected in the colon content of the IBW lambs, while three OTUs (OTU742 belonging to the class *Clostridiales*, OTU444 belonging to the family *Prevotellaceae*, and OTU447 belonging to the *Bacteroidales S24–7* gut group) were only present in the colon content of the IDW lambs (Additional file 3: Figure S2b).

## Discussion

The early life of young ruminant animals provides a unique window to potentially program the development of the rumen microbiota [19, 33, 34]. Inoculation of adult rumen microbiota to young ruminants was thought to be an effective approach to reprogram. However, earlier studies only focused on the impacts of inoculation on the composition of the rumen microbiota and the growth of young lambs [35, 36]. In the present study, we performed inoculation both before and during weaning to determine if it could promote the development of the microbiota in both the rumen and the colon and alleviate the weaning stress, which can profoundly decrease the growth performance. The potential impacts on the growth of all the intestinal segments and the colonic microbiota were also evaluated. Overall, the inoculation, either before or during weaning, had little effect on the growth of all the animals. This is in general agreement with the findings of the study using Merino lambs that were also inoculated with mature rumen fluid during suckling [37]. In a study where mature rumen fluid was inoculated into weaned lambs for seven consecutive days, apparently digestibility of dry matter (DM), crude protein (CP), fat, neutral detergent fiber (NDF), and acid detergent fiber (ADF) was improved over the course of 28 days, but those improvements were not translated into an increase in DM intake or ADG [36]. As evaluated by the empty weight and length of the intestinal segments, the inoculation, either before or during weaning, also had little effect on the growth of the rumen or intestinal tracts. The results of the present study and the two studies mentioned above collectively suggest that although the rumen microbiota may have plasticity in its development and its composition can be altered, the microbial changes do not necessarily result in significant improvements in animal growth performance [37]. The limited benefits of rumen fluid inoculation might be attributed to the lack of a suitable environment in the underdeveloped rumen of young lambs and claves. First, the inoculated rumen microbiota might bypass the rumen rapidly through the esophageal groove. Second, young lambs and calves consumed no or little solid feed, to which the rumen microbiota of adult ruminants is adapted. In addition, the duration and frequency of inoculation might not be adequate. Nevertheless, anaerobes predominant in rumen microbiota of adult ruminants could be found in the rumen of lambs at 2 days of age [25], and both the diversity and density of rumen bacteria started to stabilize and resemble that of mature rumen by week 3–4 after birth [26, 38]. To examine the effects of ruminal microbiota transplantation on the lamb performance during the weaning period, 4 weeks (7 days after starter feed intake) was chosen as the experimental period. However, it might take longer for the benefits of rumen fluid inoculation to be manifested. Thus, the age of the animals, duration and frequency of rumen fluid inoculation, inocula used, time of sampling and growth evaluation, and the long-term effects should be carefully considered in future studies to further explore rumen fluid inoculation as a potential approach to manipulate the development of the rumen microbiota and its functional impact.

Several inoculum-predominant OTUs belonging to the genera *Moryella*, *Tyzzerella* 4, and *Succinicalsticum* were established in the rumen of the IBW lambs. These OTUs established themselves in the rumen of the IBW lambs likely due to the inoculation. The use of a diverse rumen microbiota, instead of one or few single species that have been maintained under and adapted to laboratory conditions, might have increased the possibility of successfully introduce rumen microbes to the rumen. This premise is corroborated by the study of Ishaq et al. [39]. Fewer inoculum-predominant OTUs were established in the rumen of the IDW lambs than of the IBW lambs, which suggests that the rumen before weaning is more acceptive to inoculated rumen bacteria than that during weaning. This observation is consistent with the mechanism of colonization resistance and indicates that the rumen microbiota increases its resistance to colonization of inoculated bacteria as the animals grow. Several genera, including *Succiniclasticum* and uncultured *Bacteroides S24–7* family, increased in the rumen of both the IBW and the IDW lambs, and *Moryella* was increased in the rumen of the IBW lambs, suggesting that the inoculation with rumen fluid facilitated the colonization of these taxa. This premise is consistent with the finding of Abecia et al. [40] which showed that both *Succiniclasticum* and *Moryella* increased in the rumen of baby goats fed with their mothers compared to those separated from their mothers. Species belonging to the genus *Succiniclasticum* can utilize succinate producing propionate [41–43]. Shabat et al. [44] showed that cattle with enriched propionate-producing rumen bacteria and genes encoding propionate production could achieve efficient feed utilization. Species belonging to *Moryella* were also predominant in calves with a low residue feed intake when they were fed with low energy density feed [45]. Inoculum-predominant OTU belonging to uncultured *Bacteroides S24–7* family was found in the rumen of both the IBW (2 in 12 OTUs) and the IDW lambs (3 in 10 OTUs), and a greater predominance was found in the rumen of the IDW lambs. Starter feed intake might have facilitated its establishment because it can degrade complex glycan such as hemicellulose and pectin in the GI tract of homeothermic animals [46]. Researches also reported after high grain intake or subacute rumen acidosis challenge with a high grain diet, *S24–7* family in rumen decreased, intake of the starter contain forage might facilitate the colonization of this taxon, while starter introduced more plant fiber rather than only simple carbohydrates to the rumen of lambs [47, 48]. In another finding from Abecia et al. [40], *S24–7* was also reported to be more abundant in the rumen of naturally fed kids, while the family *Ruminococcaceae* and *Prevotellaceae* were gradually colonized in both twin kids. Our results indicated family *Ruminococcaceae* and genus *Prevotella* 1 were predominated in the inoculum microbiota and the rumen of all the lamb groups, suggesting that they might naturally colonize the rumen of the young lambs, while *Succiniclasticum*, *Moryella*, and *S24–7* might be transferred from ewe to their kids (through direct contact in other research or inoculation in our research). However, it should be noted that dead cells, VFAs, or soluble chemicals in rumen fluid can also impact the colonization of rumen bacteria. Indeed, lipopolysaccharides were shown to alter some bacterial populations in the rumen [49]. Muscato et al. [50] had also demonstrated positive growth performance outcomes of calves after inoculation with fresh, autoclaved, or centrifuged rumen fluid. It is speculative but the enhanced colonization of *Succiniclasticum* and *Moryella* in the rumen of the IBW lambs might potentially improve their propionate-producing potential and then feed efficiency. Again, long-term experiments are needed to test this premise and better controls were needed to elucidate the underpinning mechanisms.

The alteration of rumen bacterial genera in the rumen of the IBW and the IDW groups could be attributed directly to colonization of some bacteria which were present in the inocula and indirectly to the interactions between the established bacteria after inoculation and naturally acquired one such as *Prevotella* or *Ruminococcaceae* (both of which are the major bacterial genera in the rumen). The inocula and the rumen of the control lambs had distinct microbiota composition, reflecting the different stages of the rumen microbiota development. The LEfSe analysis revealed 11 differential OTUs, most of these OTUs belong to the phylum *Bacteroidetes* (i.e., *Prevotella* 1, *Rikenelaceae RC9* gut group and *Bacteroidales*), were generally the most predominant rumen bacteria of different adult ruminants [51], and the abundance of phylum *Bacteroidetes* was proved to increase with age [27, 28]. Thus, these OTUs could be considered as potential biomarkers of different development stages of the rumen microbiota. Interestingly, the rumen microbiota of the IBW lambs had more positive co-occurrences between the established OTUs and the biomarker OTUs, suggesting beneficial effects of inoculation on the assemblage and development of the rumen microbiota in the young lambs. Anaerobiosis is vital for the colonization of obligate anaerobes, and it is initially created by the primary colonizers that are facultatively anaerobic and can consume the oxygen in the rumen. Some of the positive co-occurrence interactions observed between anaerobic bacteria, such as the family *Ruminococcaceae*, the order *Bacteroidales*, and the genera *Pseudobutyrivibrio* and *Prevotella*, especially in the rumen of the IBW lambs, could facilitate the colonization of the rumen of young lambs by some of the inoculated bacteria, a premise in agreement with the findings of Jami et al. [27]. Species of *Moryella* [52] could produce VFAs using simple carbohydrates generated from fiber degradation by some rumen bacteria such as the *Ruminococcaceae* NK4A214 group, which was one of the major taxa in the inocula. *Moryella* species were also predominant in the rumen of forage-fed lambs, which was thought to be attributed to the digestion of fiber by fibrolytic microorganisms [53]. In this case, the rich supply of simple carbohydrates in the milk replacer (MR) might facilitate the establishment of (OTU232) *Moryella* before weaning, and its ability to utilize simple carbohydrates might explain the co-occurrence with some OTU biomarkers (such as OTU67 belongs to *Ruminococcaceae* NK4A214) in the rumen of IBW lambs after inoculation. In the human GI microbiota, bacteria exhibiting negative co-occurrence tend to have similar functions as revealed by smaller phylogenetic and functional distances, while bacteria showing positive co-occurrence do not [54]. Interactions between microbes with complementary functions can potentially affect the colonization progress of the rumen and the colon after inoculation and increase the robustness of their microbial ecology [55]. Indeed, bacteria inoculated into the human gut could better colonize and persist if they can occupy the unfilled niches therrein [56, 57]. The negative relationships between the established bacterial OTUs and the inoculum-predominant OTUs seen in the rumen of the control lambs may suggest that without the intervention, the rumen microbiota after weaning could hinder the colonization of some taxa from the inoculum, which further support the effective intervention of rumen microbiota before weaning.

With an under-developed rumen, young ruminants digest the ingested feed primarily in the small intestines. But the colonic microbiota is also important as it ferments undigested carbohydrates and protein, producing VFAs to stimulate the development of intestinal epithelium and reducing pathogen infection by either directly competing with pathogens or indirectly stimulating immune responses [58–60]. Overall, the colonic microbiota was affected by the inoculation to a less extent than the rumen microbiota. This is not surprising given that the inoculum was collected from the rumen of adult sheep. The limited effect on the lamb scouring were also consistent with the limited impacts on the colonic microbiota. Several inoculum-predominant genera were significantly increased in the colon of the IDW lambs, but not the IBW lambs. These results are in general agreement with the successful colonization of some rumen bacteria in the colon of calves when they started consuming solid feed, and the number of taxa shared between the rumen and the colon increased as calves grew [61]. It is interesting to note that *Veillonella* was enriched in the colon of both the IBW and the IDW lambs in our study. Species of *Veillonella* are major bacteria in the GI tract, and they can produce lactate or succinate for VFAs production [62]. The increase in *Veillonella* in response to the inoculation can be beneficial to the lambs. Fecal samples were not collected in our study. Future research is warranted to evaluate how the inoculation of young lambs affects fecal microbiota alteration and health, especially in lambs or claves that suffer from diarrhea.

As in other rumen inoculation studies, in the present study, we could not obtain definitive evidence if all the inoculation-promoted bacteria in the inoculated lambs were directly attributed to the inoculation for several reasons. First, the inoculated lambs (including the mock-inoculated control lambs) and the adult sheep donors of the inocula were kept on the same farm although without direct contact. Rumen bacteria from the adult sheep could be transmitted to the young lambs through airborne transmission, but it had not been proved yet. Second, the nutritional and chemical factors of the inocula could also affect the bacterial colonization as we have discussed above. Furthermore, animal-to-animal variation is inherent to the rumen and the GI microbiota, and such variation introduced confounding effects during transfaunation [6]. Thus, we used triplet lambs to minimize confounding genetic and maternal effects on the rumen and the colon microbiota [63], but triplet blocks still exhibited some variation in the rumen and the colon microbiotas. The difference in inoculation (including the mock inoculation of the control lambs) timing (the first inoculation of the IDW lambs was 14 days after that of the control lambs) and frequency (6, 4, and 2 times for the control, the IBW, and the IDW lambs, respectively) could also have potentially affected the development of the rumen and the colon microbiotas. It is extremely difficult to determine if specific bacteria present in an inoculum have successfully colonized the rumen or the GI tracts after inoculation or microbiota transplantation. Sequence markers (i.e., single-nucleotide variant) were applied in human research for tracking the colonization of foreign microbes in infants [64]. However, without specially genetically tagging the bacteria of interest, using germ-free animals, or animals in total isolation, the detection of a bacterium in both the inocula and the inoculated animals or humans cannot be exclusively attributed to the inoculation. Because genetically tagging can alter the ecological fitness of bacteria and only available for a few bacterial taxa such as *Escherichia coli* [65], it has not been used to trace inoculated bacterial community. The above potential confounding effects can be eliminated but can be minimized by isolating the inoculated lamb groups to lower the potential of airborne transmission of rumen bacteria, using the same inoculation timing and frequency, and including a control group inoculated with autoclaved rumen fluid. Apart from the rumen, the potential effects on gut health of young ruminants should also be evaluated.

## Conclusions

Inoculation with the rumen fluid from adult sheep helped the establishment of new bacterial features (such as increased predominant and positive co-occurrence) in the rumen of young lambs, and the timing of the inoculation affected the features introduced. *Moryella* and *Succiniclasticum* were among the rumen bacteria that significantly increased in the rumen of the young lambs after inoculation, and they could be potential targets for rumen microbial inoculation. Colonic microbiota was affected to a lesser extent than the rumen microbiota, and inoculation together with consumption of solid feed expanded the effects of the inoculation. Inoculation, especially early inoculation before weaning, also increased the co-occurrence interaction between established taxa after inoculation (especially *Moryella*) and some of the predominant taxa present in the inocula. Taken together, the rumen microbiota of young lambs has considerable plasticity for manipulation by inoculation with rumen fluid from adult sheep to potentially improve animal growth. Future research needs to consider the factors that can potentially affect the outcome (such as donor of rumen fluid, timing, dose, frequency, and duration of inoculation, the length of the experiment) and expand the outcomes to be examined (such as epithelium, immune response, blood metabolites, gut health).

## Methods

### Preparation of inoculum and animal experiment

A total of five adult female Hu sheep served as the donors of fresh rumen fluid throughout the inoculation experiment. We used five sheep as the donors to achieve a composite inoculum that has a broader representation of the rumen microbiota among sheep. These five sheep with bodyweights of 36.04 ± 0.27 kg were each fed a commercial concentrate diet 200 g/d twice daily and allowed to have free access to water and Chinese wildrye as forage. Fresh rumen fluid was collected from each donor sheep before morning feeding via oral tubing [66] on each inoculation day. Pooled rumen fluid (equal volume) was filtered through four layers of cheesecloth to remove large feed particles under a constant flux of CO_2_ and was kept in a closed bottle at 39 °C in a water bath until inoculation within 30 min. Each inoculum was subsampled (six aliquots, referred to as I8, I9, I15, I16, I22, and I23, with the number corresponding to the days of collection and inoculation) and preserved at − 80 °C for DNA extraction.

Fifteen Hu lamb triplets (birth body weights: 2.63 ± 0.08 kg) borne of five ewes within a three-day window were procured from a local lamb stock farm immediately after birth. All the lambs were bottle-fed the colostrum during the first 3 days and then transported to the experimental farm of Zhejiang University (a less than two-hour trip). The triplet lambs were allocated into three inoculation groups with each group randomly receiving one of the triplets, and the three lambs from the same triplet were considered as a block. Similar to the study by De Barbieri et al. [35], inoculation of rumen fluid was performed via a soft silicon tube, with a 20 cm insertion depth. The tube was inserted into the bottom of the rumen, which had been confirmed using one extra lamb before inoculation. Two of the lamb groups were repeatedly inoculated with 20 ml of the composite inoculum each time either before weaning (IBW, 4 inoculations) or during weaning (IDW, 2 inoculations), while the third group was mock-inoculated with 20 ml normal saline (6 inoculations) and served as the control (C) (Fig. 1). All the lambs were housed individually and separated by iron fence (lamb was considered as the experiment unit) and fed the same commercial milk replacer (MR; DM: 95.29%, CP: 23.77%, EE: 13.10%, NDF: 6.57%, ADF: 3.57%) three times daily at 07:00, 12:00, and 19:00. All the lambs consumed their daily MR allowance throughout the experiment. To meet their increasing nutritional requirements, each lamb was fed an increasing amount of the MR, from 240 ml/d at d 1 of the experiment (4 days of age) to 720 ml/d at d 21 (a daily increment of 24 ml). From day 22 onward, the lambs were weaned off the MR within 4 days. During weaning, a commercial starter consisting of corn and soybean meal pelleted with 20% alfalfa (concentrate: forage = 80: 20; DM: 90.12%; CP: 16.52%; NDF: 29.35%; ADF: 12.57%) was offered ad libitum to the lambs while milk replacer was reduced from 720 ml/d to 0 ml/d (a daily decrement of 180 ml) to stimulate starter feed intake. The fecal fluidity of each lamb was scored daily from day 8 to 28 based on a 5-point-scale [67]. Lambs were considered suffering from scouring when they showed a fecal score > 3.

### Growth performance, sampling and analysis, and GI morphology measurement

In the last 2 days of every week, all the lambs were weighted before morning feeding to calculate the ADG. Accumulated starter intake between days 21 and 28 was also recorded. All the lambs were euthanized on day 28 at 10:00 am (3 h after morning feeding), and 50 ml of rumen content were immediately collected into a 50-ml sterile plastic tube from each lamb. Aliquots of 2 ml of each rumen content were stored at − 20 °C until DNA extraction. Rumen pH was measured immediately after sampling using a pH meter. All the rumen content samples had little liquid so they were individually re-suspended in phosphate buffer saline (PBS), mixed by vortexing, and clarified using centrifugation at 13,000×g for 10 min to prepare the supernatant for determination of the concentrations of VFA using gas chromatography [68] and NH_3_-N using a colorimetric method [69]. Similarly, colon content was also sampled, preserved for DNA extraction, and subjected to VFA and NH_3_-N analysis.

After sampling the content, the length of each GI segment was measured after it was gently coiled around two plastic pegs (50 cm apart) to avoid stretching as Baldwin has suggested [70]. Then, all GI tract segments were separated gently and emptied after cutting open longitudinally. The empty rumen, reticulum, omasum, abomasum, small intestine, cecum, and colon were weighted after rinsing with sterile PBS. The weight of each GI segment was divided by the body weight (BW) of the lamb to minimize individual variance.

### DNA extraction, sequencing, and analysis of 16S rRNA genes

Metagenomic DNA was extracted using the CTAB (cetyltrimethyl ammonium bromide) method [71] after the samples were washed once with TN150 buffer (10 mM Tris-HCl [pH 8.0], 150 mM NaCl) as described by Li et al. [72]. The microbiota of the rumen and the colon was analyzed using 2 × 250 paired-end sequencing of amplicons of the hypervariable V3-V4 region [73] on a HiSeq platform.

Sequence data processing and analysis were performed using QIIME [74] essentially as done previously [68] with slight modifications of several parameters. Briefly, adapters and primers were trimmed off, and sequencing reads with a Q < 30 were filtered out. Potential chimeric sequences were identified and removed following joining the two paired reads using UCHIME [75]. On average 39,281 quality-checked sequences resulted from 88,783 raw sequences per sample and were used in further analysis. The quality-checked sequences were de novo clustered into operational taxonomic units (OTUs) at 97% similarity using Usearch [76]. Then, representative sequences defined by abundance from individual OTUs were aligned against the SILVA 16S reference dataset (SILVA version 123) using PyNAST [77, 78] and taxonomically assigned using the Classifier of RDP [79]. For the uncultured taxa, the nomenclatures of the SILVA database were used. α-Diversity measurements, including the observed number of OTUs, Chao1 richness estimate, and Shannon diversity index, were calculated using QIIME.

To infer the microbes that had been established in the rumen of the inoculated lambs by the inoculation, the genera and OTUs that significantly differed in relative abundance between the inoculum and the rumen contents of control lambs were identified. Of these, the ones more predominant in the inoculum than in the rumen of the control lambs were referred to as “inoculum-predominant genera or OTUs” for ease of reference. A similar comparison between the control and IBW or IDW groups was made to infer the genera and OTUs that had been established probably through the inoculation. Specifically, the OTUs which were detected in both the inoculum and the rumen of the IBW or the IDW lambs (more than three individuals, including three) but not (less than three individuals) in the rumen of the control lambs were considered the OTUs that had been successfully established in the rumen of the young lambs by the inoculation. The distribution of the inoculum-predominant genera (or OTUs) in the rumen samples of the lambs (the colonic samples were also included for comparison) was visualized using a heatmap after the average relative abundance of each genus and OTU was divided by the standard deviation to minimize the divergence between different taxa.

### Statistical analysis

Normal distributed data was tested using two-way ANOVA in R (Version 3.4.1) based on a randomized block design without interaction between block and inoculation. The data of GI weight and length measurements were abnormally distributed, they were normalized against bodyweight prior to analysis. Frequency counts data were analysed using Pearson’s Chi-square test. The permutation ANOVA test based on randomized block design was used to identify the significant difference of taxa and other abnormally distributed data between the lamb groups with the lmPerm package in R. Individual lambs from the same triplet were considered as a block. Significant difference was declared at *P* ≤ 0.05, while trend was considered at 0.05 < *P* ≤ 0.1. Post-hoc test was done to make multiple comparisons using the pairwise permutation test in the rcompanion package in R (Version 1.13.4) or Tukey pairwise test in the emmeans package in R. For the ruminal OTUs data, the non-parametric Kruskal-Wallis test was used to determine the differences between the control lambs and the inoculum (including α-diversity comparison), and the non-parametric two factor Friedman rank test without replication was used to determine the difference between each of the inoculation groups and the control group using the agricolae package in R. β-Diversity based on Bray-Curtis dissimilarity of the inoculum samples and the ruminal samples of the lambs were analyzed using PERMANOVA analysis [80] and adonis in the vegan package of R and the phyloseq package in R [81]. Pairwise comparisons were made using permutation ANOVAs adjusted by false discovery rate. A distance matrix was then ordinated by the non-metric dimensional scaling (NMDS) analysis with a 0.1225 stress value for both dimensions.

The linear discriminant analysis effect size (LEfSe) analysis [82] was used to estimate the effect size of OTUs that attributed to the inocula and the rumen samples of the control lambs so that biomarkers of inocula (proxies of the rumen microbiota of the adult sheep donors) can be identified. The OTUs with an LDA score > 2 in the inoculated lambs were considered as probable biomarkers of the rumen microbiota of the adult sheep donors. The co-occurrence of the biomarkers and the established OTUs in the lamb rumen was discriminated using network analysis based on Spearman correlation. Correlation matrices were calculated using the Hmisc package in R, and significant correlations with a coefficient value > |0.8| were used to build the networks that were visualized, and network statistics degree and average betweenness of nodes were calculated in Gephi [83].

## Supplementary information

**Additional file 1: Table S1.** Effects of rumen fluid inoculation on scouring days of the lambs. **Table S2.***P*-value matrix of pairwise adonis analysis of the inocula in different inoculation time**. Table S3.***P*-value matrix of pairwise adonis analysis of the rumen and the colon microbiota**. Table S4.** Ruminal and colonic genera that differed significantly in relative abundance between the IBW and the control lambs**. Table S5.** Ruminal and colonic genera that differed significantly in relative abundance between the IDW and the control lambs.

**Additional file 2: Figure S1.** Major bacterial taxa with a relative abundance > 0.5%. a: major bacterial taxa in the inoculum and the rumen of the lambs. b: major bacterial taxa in the colon content of the lambs. C: Control; IBW: Inoculation before weaning; IDW: Inoculation during weaning; I: Inoculum. T: triplet.

**Additional file 3: Figure S2.** An abundance heatmap showing the OTUs that were exclusively detected in the rumen of ≥3 lambs of the five IBW lambs (IBW-Unique), of ≥3 lambs of the five IDW lambs (IDW-Unique), or both the IBW and the IDW lambs (≥3 of the five lambs in each group).

**Additional file 4.** ARRIVE Guidelines Checklist.

## Data Availability

The raw DNA sequences datasets analysed during the current study are available in the NCBI Sequence Read Archive (SRA) database (https://www.ncbi.nlm.nih.gov/sra) under the accession numbers SRR7539264 - SRR7539299.

## Abbreviations

ADF: Acid detergent fiber
ADG: Average daily gain
BW: Body weight
CP: Crude protein
CTAB: Cetyltrimethyl ammonium bromide
DM: Dry matter
GI: Gastrointestinal
IBW: Inoculate before weaning
IDW: Inoculate during weaning
LDA: Linear discriminant analysis
LEfSe: Linear discriminant analysis effect size
MCP: Microbial crude protein
MR: Milk replacer
NDF: Neutral detergent fiber
NH_3_-N: Ammonia nitrogen
NMDS: Non-metric dimensional scaling
OTU: Operational taxonomic units
PBS: Phosphate buffer saline
RDP: Ribosomal Database Project
RMT: Ruminal microbiota transplantation
rRNA: Ribosomal ribonucleic acid
SARA: Subacute ruminal acidosis
VFA: Volatile fatty acids
