# Supplementary material for: Repeated inoculation with fresh rumen fluid before or during weaning modulates the microbiota composition and co-occurrence of the rumen and colon of lambs
Source: BMC Microbiol. 2020 Feb 7;20:29. doi: 10.1186/s12866-020-1716-z (PMC7006167; doi:10.1186/s12866-020-1716-z)
Supplement: Supplementary file 1 — Additional file 1: Table S1. Effects of rumen fluid inoculation on scouring days of the lambs. Table S2.P-value matrix of pairwise adonis analysis of the inocula in different inoculation time. Table S3.P-value matrix of pairwise adonis analysis of the rumen and the colon microbiota. Table S4. Ruminal and colonic genera that differed significantly in relative abundance between the IBW and the control lambs. Table S5. Ruminal and colonic genera that differed significantly in relative abundance between the IDW and the control lambs. [file 12866_2020_1716_MOESM1_ESM.pdf]

Table S1. Effects of rumen fluid inoculation on scour days of lambs

Experimental days	Counts			P-value**	
	Normal Days	Scour Days	Total	C vs IBW	C vs IDW
**Day 8-21 (n=5)**					
Control	62	8	70	1.00	0.37
IBW	62	8	70		
IDW*	56	14	70		
**Day 22-28 (n=5)**					
Control	33	2	35	0.56	0.56
IBW	34	1	35		
IDW	34	1	35		

*: IDW group had not receive any inoculation during this time

**: Significance is the pairwise comparison of Pearson's Chi-Square test between the three treatments

Table S2. P-value matrix of pairwise Adonis analysis of inoculation aliquots

**Fraction-Inoculation**	**Bray Curtis distance**		
	I8&I9*	I15&I16	I22&I23
I15&I16	0.330	-	-
I22&I23	0.330	0.330	-

*Two inoculating day as one group

Table S3. P-value matrix of pairwise Adonis analysis of the rumen and the colon microbiota

**Fraction-**	**Bray Curtis distance**						
**Inoculation**	Inoculum	RC-C*	RC-BT	RC-AT	CC-C**	CC-BT	CC-AT
RC-C	0.017	-	-	-	-	-	-
RC-BT	0.017	0.192	-	-	-	-	-
RC-AT	0.017	0.715	0.247	-	-	-	-
CC-C	0.017	0.028	0.018	0.017	-	-	-
CC-BT	0.017	0.019	0.018	0.017	0.726	-	-
CC-AT	0.017	0.017	0.017	0.017	0.923	0.488	-

*: RC- means rumen content from different inoculation groups

**: CC- means colon content from different inoculation groups

Table S4. Ruminal and colonic genera with significant relative abundance between IBW* and control lambs

Fraction	Taxa	Control	IBW	SEM	P-value
**Rumen Content**	g_*Prevotellaceae* UCG-001	0.439	1.081	0.126	0.014
	g_*Coprococcus* 1	0.012	0.055	0.013	0.014
	g_*Lachnoclostridium* 1	0.299	0.975	0.157	0.014
	g_*Moryella*	0.102	0.422	0.099	0.014
	g_*Tyzzerella* 4	0.034	0.134	0.019	0.014
	g_*Eubacterium* hallii group	0.318	0.491	0.162	0.014
	g_*Succiniclasticum*	0.051	1.108	0.313	0.014
**Colon Content**	g_*Veillonella*	0.001	0.174	0.081	0.014
	g_*Escherichia Shigella*	2.737	8.444	1.565	0.014

*: Inoculation before weaning, n=5

Table S5. Ruminal and colonic genera with significant relative abundance between IDW* and control lambs

Fraction	Taxa	Control	IDW	SEM	P-value
**Rumen Content**	g_*Bacteroidales* S24-7 group bacterium	0.010	0.105	0.036	0.014
	g_*Eubacterium* coprostanoligenes group	0.098	0.200	0.03	0.014
	g_*Erysipelatoclostridium*	0.006	0.013	0.003	0.046
	g_*Sharpea*	8.592	18.53	4.322	0.014
**Colon Content**	g_*Senegalimassilia*	0.029	0.013	0.007	0.046
	g_*Coprobacter*	0.226	1.202	0.329	0.025
	g_*Anaerofilum*	0.039	0.061	0.018	0.025
	g_*Ruminococcus* 1	0.015	0.034	0.010	0.046
	g_*Mitsuokella*	0.030	0.014	0.006	0.046
	g_*Veillonella*	0.001	0.022	0.004	0.025

*: Inoculation during weaning, n=5
