# Supplementary figures and images for: Repeated inoculation with fresh rumen fluid before or during weaning modulates the microbiota composition and co-occurrence of the rumen and colon of lambs
Source: BMC Microbiol. 2020 Feb 7;20:29. doi: 10.1186/s12866-020-1716-z (PMC7006167; doi:10.1186/s12866-020-1716-z)

**a**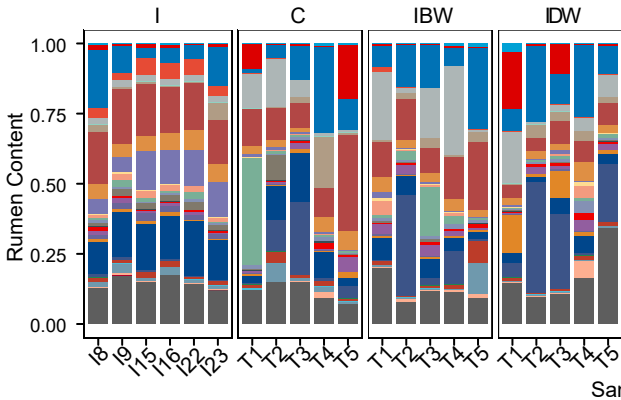**b**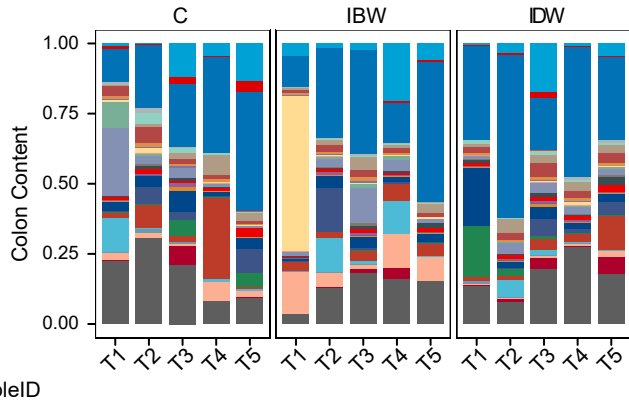**Taxa**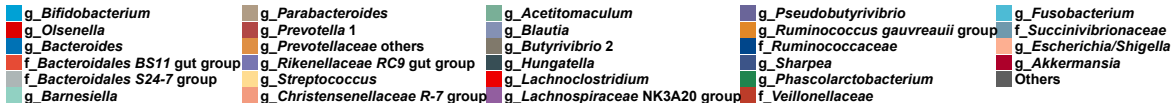

Supplement: Supplementary file 2 — Additional file 2: Figure S1. Major bacterial taxa with a relative abundance > 0.5%. a: major bacterial taxa in the inoculum and the rumen of the lambs. b: major bacterial taxa in the colon content of the lambs. C: Control; IBW: Inoculation before weaning; IDW: Inoculation during weaning; I: Inoculum. T: triplet. [file 12866_2020_1716_MOESM2_ESM.pdf]

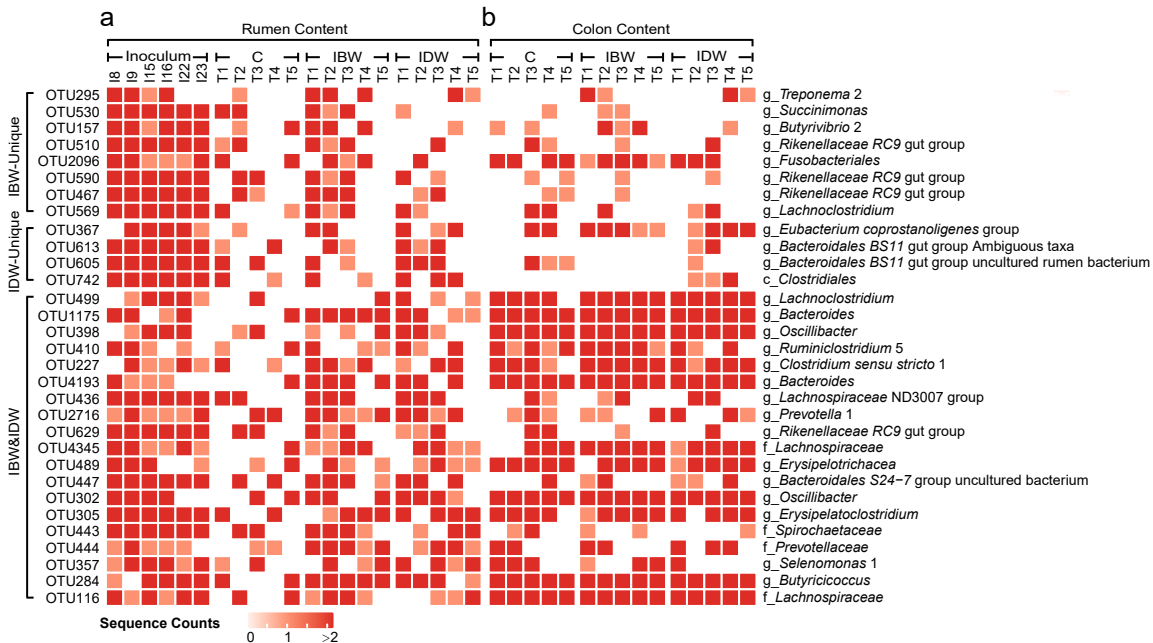

Supplement: Supplementary file 3 — Additional file 3: Figure S2. An abundance heatmap showing the OTUs that were exclusively detected in the rumen of ≥3 lambs of the five IBW lambs (IBW-Unique), of ≥3 lambs of the five IDW lambs (IDW-Unique), or both the IBW and the IDW lambs (≥3 of the five lambs in each group). [file 12866_2020_1716_MOESM3_ESM.pdf]
